# Clinical Observation and Prognostic Analysis of Patients With *Klebsiella pneumoniae* Bloodstream Infection

**DOI:** 10.3389/fcimb.2020.577244

**Published:** 2020-11-09

**Authors:** Shuguang Zhang, Ziyue Yang, Limin Sun, Zhenhua Wang, Liutao Sun, Jinli Xu, Li Zeng, Tongwen Sun

**Affiliations:** ^1^Department of General Intensive Care Unit, The First Affiliated Hospital of Zhengzhou University, Zhengzhou Key Laboratory of Sepsis, Henan Key Laboratory of Critical Care Medicine, Zhengzhou, China; ^2^Department of Intensive Care Unit, Lankao People’s Hospital, Kaifeng, China; ^3^Emergency Department, Traditional Chinese Medicine Hospital of Sui County, Shangqiu, China

**Keywords:** *Klebsiella pneumoniae*, intensive care unit (ICU), Carbapenem resistant *Acinetobacter baumannii* (CRAB), anti-bacterial agents—therapeutic use, bloodstream infection (BSI)

## Abstract

**Background and purpose:**

The clinical prognosis of Klebsiella pneumoniae(*K. pneumoniae*) bloodstream infection is poor, and the prevalence of drug-resistant bacteria makes clinical anti-infective treatment more challenging. This retrospective study evaluated the epidemiological characteristics of patients with *K. pneumoniae*, the risk factors for drug-resistant bacterial infection and death, and analyzed treatment options.

**Methods:**

Clinical data of 297 patients diagnosed with *K. pneumoniae* bacteremia between June 2014 and June 2019 were collected.

**Results:**

Intensive care unit hospitalization history, operation history, recent antibiotic use history, mechanical ventilation, and number of days hospitalized before bloodstream infection were found to be independent risk factors for drug-resistant bacterial infection. The risk of death for carbapenem-resistant *K. pneumoniae* infection was 2.942 times higher than that for carbapenem-sensitive *K. pneumoniae* infection. For extensively drug-resistant *K. pneumoniae* bacteremia patients, the mortality rate of combined anti-infective therapy was lower.

**Conclusions:**

Clinicians should pay attention to patients with high-risk drug-resistant bacteria infection and administer timely anti-infection treatment. The findings of this study may provide some suggestions for early identification and standardized treatment of patients with *K. pneumoniae* bacteremia.

## Background

Enterobacterales is a family of pathogens that cause serious infections, among which Escherichia coli and Klebsiella are the most common (Willyard, 2017). *Klebsiella pneumoniae*, a gram-negative bacillus, is the most important species of Klebsiella in the Enterobacterales family (Rodriguez-Bano et al., 2018). *K. pneumoniae* can colonize in the respiratory tract and skin, as well as become aerosolized (Denkel et al., 2019; Russo and Marr, 2019; Sequeira et al., 2020). There are a variety of clinical manifestations, including bloodstream infection (BSI), pulmonary infection, urinary tract infection, abdominal infection, and so forth (Selden et al., 1971; Dorman and Short, 2017).

Clinicians pay the most attention to BSI caused by *K. pneumoniae*. BSI involves various pathogenic microorganisms, such as bacteria or fungi, invading the blood circulation, reproducing in the blood, releasing toxins and metabolites, and inducing the release of cytokines. This can cause systemic multiple organ dysfunction syndrome (MODS), a serious systemic infectious disease, and even death. Moreover, BSI can increase the length of hospital stay, treatment costs, and complications after discharge, directly or indirectly affecting patient prognosis. It is reported that the case fatality rate of BSI is between 21% and 69% (Du et al., 2002; Kim et al., 2005; Laupland et al., 2005; Garnacho-Montero et al., 2008).

Carbapenem antibiotics are powerful β-lactam drugs for the treatment of *K. pneumoniae*-related BSIs. Once resistant to carbapenem antibiotics, clinical treatment of this type of infection is very difficult. In recent years, the detection rate of carbapenem-resistant *K. pneumoniae* (CRKP) has also increased every year (MacKenzie et al., 1997; Rahal, 2008; Capone et al., 2013; Potter et al., 2016; Willyard, 2017). The emergence of CRKP makes the anti-infective treatment encounter great challenges globally (Willyard, 2017). A study in Greece found that 10% of intensive care unit (ICU) patients had CRKP infection, and the mortality rate was four times higher than that of sensitive bacterial infections. However, clinicians are more concerned about extensively drug-resistant (XDR) *K. pneumoniae* (XDR-KP), which is resistant to more types of antibiotics than CRKP. XDR-KP is resistant to many antibiotics currently used in the clinic, except for tigecycline and polymyxin. But, emergence of high level of resistance against latest generation of tigecycline in animals and humans is concerning (He et al., 2019; Gasparrini et al., 2020). BSI caused by strain often aggravates the disease, resulting in difficult treatment and poor clinical prognosis. A large number of studies have shown that early identification and early appropriate antibiotic treatment are significantly related to the reduction of mortality (Schwaber and Carmeli, 2008; Daikos et al., 2014; Gomez-Simmonds et al., 2016; Tsala et al., 2019; Lee et al., 2020). Due to the lack of attention to high risk factors, late identification and inadequate antibacterial treatment increases mortality; thus, it is necessary to obtain cultures in the early stage of infection to determine specific pathogens, drug resistance, and reasonable antibiotic treatment.

Previous studies have discussed BSIs caused by *K. Pneumoniae* (Daikos et al., 2014; Gomez-Simmonds et al., 2016; Tsala et al., 2019; Gasparrini et al., 2020; Lee et al., 2020). However, the risk factors for drug-resistant bacterial infection and death of patients with bacteremia are still inconsistent among the different studies; thus, these risk factors still need to be further improved and verified in clinical practice (Du et al., 2002; Ben-David et al., 2012; Giannella et al., 2019; Lee et al., 2019). Early detection of risk factors and improving clinician awareness play a positive role in the prognosis of patients. Because inadequate anti-infective treatment can increase mortality, improving the identification of patients with high-risk drug-resistant bacteria is key. Once there are clinical symptoms, with respect to drug-resistant bacterial infection, whether combination therapy with anti-infective treatment is effective still needs to be further studied. Some observational studies have shown that, when confirming CRKP infection, combination therapy is related to an improvement of survival rate, but the best treatment scheme has not been determined (Gutierrez-Gutierrez et al., 2017; Doi, 2019). Therefore, we conducted a single-center retrospective study to explore the clinical epidemiology, risk factor analysis, antimicrobial analysis, and prognosis analysis of BSI patients caused by *K. pneumoniae*. We analyzed the demographic characteristics of these patients, the risk factors for drug-resistant bacterial infection and the risk factors for death. Additionally, the treatment schemes in the retrospective data were analyzed. The findings in this study are important for early detection, early treatment, and improving the prognosis of patients with BSI.

## Methods

### Research Objects

This retrospective observational study was conducted in Grade 3A Hospital, the First Affiliated Hospital of Zhengzhou University in Henan Province, China. Clinical data of patients diagnosed with *K. pneumoniae* from June 2014 to June 2019 were collected retrospectively. The inclusion criteria were as follows: 1. K*. pneumoniae* was found in at least one blood culture, and the patient met the diagnostic criteria for blood flow infection. 2. The patient was positive in multiple cultures, and the first culture was positive at the initial observation time. 3. The age of the patient was ≥18 years old. 4. The hospitalized patient had complete clinical data. The exclusion criteria were as follows: 1. The patient was discharged within 24 hours of hospitalization. 2. The patient had more than two kinds of bacterial BSIs. This study was approved by the Ethics Committee of Scientific Research and Clinical Trials of the First Affiliated Hospital of Zhengzhou University (Code 2020-KY-087).

### Definitions

The diagnostic criteria for the BSI patients included in this study can be found in the Centers for Disease Control and Prevention (CDC) guidelines (https://www.cdc.gov/nhsn/PDFs/pscManual/4PSC_CLABScurrent.pdf), including primary BSI(Primary BSI is a laboratory confirmed bloodstream infection (LCBI) where an eligible BSI organism is identified and the BSI is not secondary to a site-specific infection at another body site), secondary BSI(Secondary BSI is a bloodstream infection that is associated with a site-specific infection at another body site which may have seeded the bloodstream), and Central Line-Associated Bloodstream Infection (CLABI). The diagnostic criteria for septic shock were based on the Sepsis 3.0 guidelines jointly issued by the Society of Critical Care Medicine (SCCM) and the European Society of Intensive Care Medicine (ESCIM) in 2016 (Singer et al., 2016). The ventilator application included non-invasive ventilation and invasive ventilation. Non-invasive ventilation included mask ventilation and excluded high-flow nasal catheter oxygen therapy. Invasive ventilation included orotracheal intubation and tracheotomy connected to a ventilator. Referring to the consensus co-sponsored by the European and United States CDC in 2012 (Magiorakos et al., 2012), multidrug resistance (MDR) is defined as insensitivity to three or more types of antibiotics (including drug resistance and intermediation) within the antibacterial spectrum. XDR is defined as insensitivity to almost all types of antibiotics except one or two types of antibiotics (mainly polymyxin and tigecycline), and the determination of class resistance is the same as MDR. Pandrug resistance (PDR) is defined as insensitivity to all types of antimicrobials currently used in the clinic. Combination therapy is defined as a regimen that is treated with two or more antibiotics approved by infectious disease specialists in the course of treatment.

### Microbiology and Drug Sensitivity Test

*K. pneumoniae* was identified using the VITEK2 system (bioMèrieux, Marcy l’Etoile, France). A direct rapid drug sensitivity test was conducted using the positive blood culture bottle and the disk diffusion method. The results of the antibiotic sensitivity test were described according to the guidelines issued by the European Committee on Antimicrobial Susceptibility Testing (EUCAST).

### Clinical Data Collection

Previous cases were consulted and the data were collected as follows: General patient data (i.e., sex, age, and underlying diseases) were collected. Pre-hospitalization data, including hospitalization history, ICU hospitalization history, operation history, antibiotic use history, and glucocorticoid and immunosuppressant use history, were recorded. Past patient history was obtained, such as history of diabetes, hypertension, coronary heart disease, chronic kidney disease (CKD), et al. Clinical data before blood culture diagnosis was collected, such as inpatient department, nosocomial infection, Acute Physiology and Chronic Health Evaluation II (APACHE II) score, Sequential Organ Failure Assessment (SOFA) score, Pitt score, Charlson complication index, et al. Recent invasive procedures were also noted, such as central venous catheter (CVC) placement, temporary dialysis tube placement, arterial puncture, tracheal intubation, et al. The results of the blood culture and drug sensitivity tests were recorded. The antibiotic treatment plan and the clinical data of the patients after commencing treatment were recorded. The main outcome index was the 28-day mortality after the diagnosis of BSI, and the secondary outcome indicators included total hospital stay duration, ICU hospital stay duration, mechanical ventilation time, vasoactive drug use, acute renal failure, and MODS.

### Statistical Methods

The Windows version of SPSS 21.0 software was used for analysis (IBM SPSS Statistics, IBM Corporation, Armonk, New York). Continuous variables with a normal distribution were represented by the mean ± standard deviation (mean ± SD), and the non-normal distribution data were represented by the median [interquartile range] (Median[IQR]). In this study, only the age data had a normal distribution, and the age comparison between the two groups was analyzed using two independent sample t-tests. Data with a non-normal distribution were calculated using the Mann-Whitney U test. Categorical variables were compared using a χ^2^ test or Fisher’s exact test to compare the differences between groups. Variables with *p* < 0.05 upon univariate analysis were included in the multivariate analysis. The risk factors for drug-resistant bacterial infection and the independent predictors of 28-day mortality were determined using binomial logistic regression. The *p* value, odds ratio (OR), and 95% confidence interval (CI) of the risk factors were calculated to evaluate the intensity of the association. A *p* value less than 0.05 was considered statistically significant.

## Results

This retrospective observational study included 297 patients with *K. pneumoniae* BSI. The average age was 55 ± 16 years old, of which 202 (68%) were male patients. Among the positive blood culture results, there were 114 sensitive *K. pneumoniae* (S-KP) cases, 99 multidrug-resistant *K. pneumoniae* (MDR-KP) cases, and 84 XDR-KP cases. There were no PDR bacteria in these patients.

### Drug-Resistant Bacterial Infection Clinical Characteristics and Risk Factors

According to the different results from the drug sensitivity test, patients were divided into three groups: the S-KP group, MDR-KP group, and XDR-KP group. The demographics and clinical characteristics of the three groups are shown in **Table 1**. There was no difference in sex and age between the different groups. By comparing the S-KP group with the drug-resistant group (including MDR-KP and XDR-KP groups), we found that patients who were hospitalized within 30 days before infection (*p* = 0.001), recent history of ICU hospitalization (*p* < 0.001), and patients who have been operated on recently (*p* < 0.001) were more likely to be infected with drug-resistant bacteria. We compared the recent invasive procedures between the sensitive group and the drug-resistant group and found that the drug-resistant group had a more recent indwelling CVC (*p* < 0.001), trachea cannula (*p* < 0.001), tracheotomy (*p* = 0.001), arterial puncture (*p* = 0.001), thoracentesis (*p* = 0.001), indwelling catheter (*p* < 0.001), and gastric tube (*p* < 0.001). Despite the lack of statistical significance, there were more cases of dialysis tube placement in the drug-resistant group, suggesting that such cases require more attention from clinicians. Since fewer patients with a history of organ transplantation, corticosteroid use, or immunosuppressant use were included in the study, the clinical reference value of the comparison between the two groups was small. In the drug-resistant bacterial infection group, more patients were treated with antibiotics intravenously (*p* = 0.045). The drug-resistant group had a longer hospital stay before the diagnosis of BSI (*p* < 0.001). With respect to the ward in which the patient was admitted at the time of the BSI diagnosis, we found that there were more sensitive BSIs in the internal medicine ward (*p* = 0.001) and more resistant infections in the ICU (*p* = 0.002). When comparing basic diseases between the two groups, there were more diabetic patients in the drug-resistant group (*p* = 0.007). We also included the basic information of the patients from the day of the BSI diagnosis. From this information, we can see that the Pitt score (*p* < 0.001), acute APACHE II score (*p* < 0.001), and SOFA score (*p* = 0.006) of the drug-resistant bacteria group were higher. There were more patients with respiratory failure in the drug-resistant bacteria group (*p* = 0.007), more patients under recent ventilation (*p* < 0.001), and more patients with MODS (*p* < 0.001), hypoalbuminemia (*p* = 0.05), and septic shock (*p* < 0.001).

**Table 1:** Clinical characteristics of patients in different groups before diagnosis of bloodstream infection.

Characteristic	All patients	S-KP	MDR-KP	XDR-KP	p*
n	297	114	99	84	
Gender^∮^ (Male, %)	202 (68)	73 (64)	69 (69.6)	60 (71.4)	0.246
Age^∯^ (Years)	55 ± 16	55 ± 16	56 ± 16	53 ± 16	0.798
Hospital before^1∮^	149 (50.1)	43 (37.7)	47 (47.4)	59 (70.2)	0.001
ICU before^1∮^	113 (38)	21 (18.4)	33 (33.3)	59 (70.2)	<0.001
Surgery^2∮^	105 (35.3)	25 (21.9)	35 (35.3)	45 (53.5)	<0.001
Invasive operation^3∮^					
CVC	144 (48.4)	35 (30.7)	49 (49.4)	60 (71.4)	<0.001
Subclavian vein	89 (61.8)	26 (74.2)	25 (51)	38 (63.3)	0.081
Internal jugular vein	46 (31.9)	7 (20)	21 (42.8)	18 (30)	0.082
Femoral vein	9 (6.2)	2 (5.7)	3 (6.1)	4 (6.6)	1
Dialysis tube	46 (15.4)	12 (10.5)	14 (14.1)	20 (23.8)	0.062
Arteriopuncture	183 (61.6)	57 (50)	55 (55.5)	71 (84.5)	0.001
Trachea cannula	87 (29.2)	15 (13.1)	34 (34.3)	38 (45.2)	<0.001
Tracheotomy	32 (10.7)	4 (3.5)	14 (14.1)	14 (16.6)	0.001
Thoracentesis	16 (5.3)	0	11 (11.1)	5 (5.9)	0.001
Abdominocentesis	18 (6)	6 (5.2)	7 (7.1)	5 (5.9)	0.649
Lumbar puncture	23 (7.7)	6 (5.2)	9 (9.1)	8 (9.5)	0.207
Bone marrow aspiration	35 (11.7)	13 (11.4)	10 (10.1)	12 (14.2)	0.872
Indwelling catheter	168 (56.5)	45 (39.4)	56 (56.5)	67 (79.7)	<0.001
Indwelling gastric tube	120 (40.4)	30 (26.3)	36 (36.3)	54 (64.2)	<0.001
Organ transplantation^4∮^	9 (3)	2 (1.7)	1 (1)	6 (7.1)	0.311
Application of antibiotics^1∮^	96 (32.3)	29 (25.4)	36 (36.3)	31 (36.9)	0.045
Glucocorticoid^1∮^	18 (6)	4 (3.5)	8 (8.1)	6 (7.1)	0.146
Immunosuppressant^1∮^	22 (7.4)	7 (6.1)	9 (9.1)	6 (7.1)	0.510
Hospital infection^∮^	162 (54.5)	58 (50.8)	54 (54.5)	50 (59.5)	0.316
Hospital days before infection^∰^	10 [4,20]	6 [3,11]	11 [5,23]	16 [9,24]	<0.001
Inpatient department^5^					
Internal Medicine	106 (35.6)	54 (47.3)	34 (34.3)	18 (21.4)	0.001
Surgery Ward	64 (21.5)	24 (21)	26 (26.2)	14 (16.6)	0.87
ICU	127 (42.7)	36 (31.5)	39 (39.3)	52 (61.9)	0.002
Pitt bacteremia score^∰^	2 [0,4]	1 [0,2]	2 [0,5]	3 [1,6]	<0.001
Charlson comorbidity index^∰^	3 [2,4]	3[2,5]	3 [2,4]	2 [1,4]	0.443
Basic diseases^∮^					
Hypertension	86 (28.9)	31 (27.1)	26 (26.2)	29 (34.5)	0.579
Diabetes	69 (23.2)	17 (14.9)	25 (25.2)	27 (32.1)	0.007
Chronic renal failure	47 (15.8)	19 (16.6)	10 (10.1)	18 (21.4)	0.754
Liver insufficiency	47 (15.8)	17 (14.9)	14 (14.1)	16 (19)	0.734
Solid Organ Cancer	36 (12.1)	14 (12.2)	14 (14.1)	8 (9.5)	0.947
Blood disease	40 (13.4)	16 (14)	11 (11.1)	13 (15.4)	0.821
Coronary heart disease	58 (19.5)	21 (18.4)	16 (16.1)	21 (25)	0.704
Respiratory failure^∮^	60 (20.2)	14 (12.2)	13 (13.1)	33 (39.2)	0.007
Hypoalbuminemia^∮^	90 (30.3)	27 (23.6)	31 (31.3)	32 (38)	0.05
White blood cell^∰^	9 [5.5,14.4]	8.2 [4.8,13.5]	8.2 [5.5,14.1]	10.5 [6.3,16.4]	0.339
Alanine aminotrans^∰^	30 [16,61]	33 [17,60]	30 [17,59]	27 [16,66]	0.329
Creatinine^∰^	69 [49,106]	69 [50,90]	62 [48,95]	85 [53,171]	0.94
Septic shock^∮^	63 (21.2)	11 (9.6)	20 (20.2)	32 (38.1)	<0.001
MODS^∮^	48 (16.1)	7 (6.1)	14 (14.1)	27 (32.1)	<0.001
APACHEII^∯^; score^∰^	11 [6,18]	8 [5,14]	12 [7,17]	17 [9,22]	<0.001
SOFA score^∰^	4 [2,7]	3 [2,6]	4 [2,7]	7 [3,10]	0.006
Mechanical ventilation^∮^	95 (31.9)	12 (10.5)	36 (36.3)	47 (55.9)	<0.001

^1^30 days before this hospitalization; ^2^90 days before this hospitalization; ^3^30 days before positive blood culture; ^4^Before this hospitalization (no matter how long ago); ^5^The in-patient department on the day of blood sampling and examination; ^∮^ n (n%); ^∯^ Mean ± SD; ^∰^ Median[IQR]

n sample size, S-KP sensitive K. pneumoniae, MDR-KP multidrug resistant K. pneumoniae, XDR-KP extensively drug resistant K. pneumoniae, ICU intensive care unit, CVC central venous catheter, MODS multiple organ dysfunction syndrome, APACHE acute physiology and chronic health evaluation, SOFA sequential organ failure assessment.

*Single factor comparison: The comparison of susceptibility (n=114) and drug resistance (n=183), in which drug resistance includes MDR-KP and XDR-KP. Chi-Square Test is used to calculate the two-category variables, t-test is used to calculate the normal distribution variables, and Mann-Whitney U Test is used to calculate the variables with non-normal distribution and uneven variance.

Univariate analysis showed that recent hospitalization history, ICU hospitalization history, operation history, invasive operation history, antibiotic application history, hospitalization time, the hospitalization ward, scores, respiratory failure, MODS, and septic shock were the risk factors for *K. pneumoniae* BSI. After adjusting the hospitalization time and scores using binary logistic regression, we found that ICU hospitalization history (OR=2.32, 95% CI 1.22–4.4, *p* = 0.01), surgical history (OR=2.33, 95% CI 1.26–4.32, *p* = 0.007), recent antibiotic use history (OR=2.02, 95% CI 1.1–3.74, *p* = 0.024), mechanical ventilation (OR=3.3, 95% CI 1.56–6.97, *p* = 0.002), and days of hospitalization before BSI were independent risk factors for drug-resistant bacterial infection. The results of the multivariate analysis are shown in **Table 2**. By dividing the hospitalization time before infection into three groups (1 week, 2 weeks, and more than 2 weeks), we found that patients with more than 2 weeks of hospitalization before BSI were 4.34 (2.21–8.55) times more likely to be infected with drug-resistant bacteria than those who were hospitalized within one week.

**Table 2:** Logistic regression analysis of risk factors of drug-resistant *K. pneumoniae* bloodstream infections.

Characteristics	OR (95% CI)	*p*
History of ICU hospitalization		
None	1	
Have	2.32 (1.22–4.4)	0.01
History of surgery		
None	1	
Have	2.33 (1.26–4.32)	0.007
History of application of antibiotics		
None	1	
Have	2.02 (1.1–3.74)	0.024
Mechanical ventilation		
None	1	
Have	3.3 (1.56–6.97)	0.002
APACHE II score		
≤10	1	
>10	1.9 (1.06–3.42)	0.031
Hospitalization days before bloodstream infection		
≤7 days	1	
>7 days and ≤14 days	1.77 (0.88–3.55)	0.11
>14 days	4.34 (2.21–8.55)	<0.001

OR odds ratio, CI Confidence interval, ICU intensive care unit, APACHE Acute Physiology and Chronic Health Evaluation.

### Risk Factors for Death in Patients With *K. pneumoniae* BSI

Taking death within 28 days after BSI diagnosis as the main end point, the results showed that 127 cases died and 170 cases survived. The 28-day mortality rate of patients with *K. pneumoniae* BSI was 42.8%. We compared patients with different clinical outcomes, and the results obtained by univariate analysis are shown in **Table 3**. As with the risk factors of drug-resistant bacterial infection, recent hospitalization history (*p* = 0.029), ICU hospitalization history (*p* < 0.001), and surgical history (*p* = 0.026) occurred more frequently in the death group. The comparison of invasive operation between the survival group and the death group showed that patients with an indwelling CVC (*p* < 0.001), an indwelling dialysis tube (*p* < 0.001), arterial puncture (*p* < 0.001), trachea cannula (*p* < 0.001), tracheotomy (*p* = 0.022), thoracentesis (*p* = 0.007), an indwelling catheter (*p* = 0.001), and a gastric tube (*p* < 0.001) had a higher risk of death. When comparing previous basic diseases between the two groups, there were more patients with diabetes (*p* < 0.001), chronic renal failure (*p* = 0.004), and coronary heart disease (*p* = 0.006) in the death group. From the comparison of clinical characteristics before the BSI diagnosis, patients with respiratory failure (*p* = 0.001), ventilator use (*p* < 0.001), hypoalbuminemia (*p* < 0.001), septic shock (*p* < 0.001), and MODS (*p* < 0.001) predicted a worse clinical outcome. At the same time, we observed that the APACHE II score (*p* < 0.001) and SOFA score (*p* < 0.001) were higher in the death group. In the death group, the hospitalization days before BSI were relatively longer (8 [4,18] days versus 14 [5,22] days, *p* = 0.015).

**Table 3:** Comparison of the basic condition of patients between 28-day survivors and non-survivors (n,%).

Characteristics	Survivors (n = 170)	Non-survivors (n = 127)	*p*
Gender (male, %)^∫^	116 (68.2)	86 (67.7)	0.924
Age (Years, Mean ± SD)^∬^	54 ± 16	55 ± 15	0.625
Hospital before^∫^	76 (44.7)	73 (57.4)	0.029
ICU before^∫^	39 (22.9)	74 (58.2)	<0.001
Recent surgical history^∫^	51 (30)	54 (42.5)	0.026
Invasive operation^∫^			
CVC	57 (33.5)	87 (68.5)	<0.001
Subclavian vein	35 (61.4)	54 (62.1)	0.936
Internal jugular vein	18 (31.6)	28 (32.2)	0.939
Femoral vein	3 (5.3)	6 (6.9)	0.692
Dialysis tube	14 (8.2)	32 (25.2)	<0.001
Arteriopuncture	89 (52.4)	94 (74)	<0.001
Trachea cannula	21 (12.3)	66 (51.9)	<0.001
Tracheotomy	9 (5.2)	23 (18.1)	0.022
Thoracentesis	4 (2.3)	12 (9.4)	0.007
Abdominocentesis	8 (4.7)	10 (7.9)	0.258
Lumbar puncture	10 (5.9)	13 (10.2)	0.165
Bone marrow aspiration	18 (10.6)	17 (13.4)	0.459
Indwelling catheter	82 (48.2)	86 (67.7)	0.001
Indwelling gastric tube	48 (28.2)	72 (56.7)	<0.001
Organ transplantation^∫^	4 (2.3)	5 (3.9)	0.656
Application of antibiotics^∫^	59 (34.4)	37 (29.1)	0.310
Glucocorticoid^∫^	9 (5.2)	9 (7.1)	0.522
Immunosuppressant^∫^	12 (7.1)	10 (7.8)	0.791
Hospital infection^∫^	94 (55.2)	63 (53.5)	0.764
Hospital days before infection^∭^	8 [4,18]	14 [5,22]	0.015
Pitt bacteremia score (median [IQR])^∭^	2 [0,4]	2 [0,5]	0.577
Charlson comorbidity index (median [IQR])^∭^	3 [2,5]	3 [1,4]	0.277
Basic diseases^∫^			
Hypertension	47 (27.6)	39 (30.7)	0.565
Diabetes	26 (15.2)	43 (33.8)	<0.001
Chronic renal failure	18 (10.5)	29 (22.8)	0.004
Liver insufficiency	21 (12.3)	26 (20.4)	0.058
Solid Organ Cancer	23 (13.5)	13 (10.2)	0.390
Blood disease	20 (11.7)	20 (15.7)	0.320
Coronary heart disease	24 (14.1)	34 (26.7)	0.006
Respiratory failure^∫^	23 (13.5)	37 (29.1)	0.001
Hypoalbuminemia^∫^	34 (20)	56 (44)	<0.001
Drug resistance (S-KP, %)^∫^	88 (51.7)	26 (20.4)	<0.001
Drug resistance (MDR-KP, %)^∫^	56 (32.9)	43 (33.8)	0.868
Drug resistance (XDR-KP, %)^∫^	26 (15.2)	58 (45.6)	<0.001
Septic shock^∫^	15 (8.8)	48 (37.7)	<0.001
MODS^∫^	9 (5.2)	39 (30.7)	<0.001
APACHEII score^∭^	7 [4,11]	18 [13,23]	<0.001
SOFA score^∭^	2 [2,4]	8 [5,11]	<0.001
Mechanical ventilation^∫^	23 (13.5)	72 (56.6)	<0.001

^∫^Use Chi-Square Test to verify; ^∬^Two groups were compared with independent t-test; ^∭^Use Mann-Whitney U Test to verify.

SD Standard deviation, ICU intensive care unit, CVC central venous catheter, IQR inter-quartile range, MODS multiple organ dysfunction syndrome, APACHE acute physiology and chronic health evaluation, SOFA sequential organ failure assessment, S-KP sensitive K. pneumoniae, MDR-KP multidrug resistant K. pneumoniae, XDR-KP extensively drug resistant K. pneumoniae.

We compared the results of the drug sensitivity test between the survival group and the death group, and the proportion of S-KP patients in the survival group was higher, while the proportion of XDR-KP patients in the death group was higher. There was no significant difference in the distribution of MDR-KP infections between the two groups. The drug-resistance of patients in the survival group and the death group were compared one by one, as shown in **Table 4**. It was observed that the proportion of drug resistance to penicillins, cephalosporins, and carbapenems in the death group was statistically significantly higher than that in the survival group. According to the clinical data from the day of BSI diagnosis to before discharge, the risk of accident was higher in the 28-day death group. Patients requiring ventilator support, continuous renal replacement therapy (CRRT), MODS, acute renal failure, transfer to ICU, and uncleared bacteria after blood culture diagnosis predicted a poor prognosis (see **Table 5**).

**Table 4:** Comparison of drug resistance between 28-day survivors and non-survivors (n,%).

Antibacterial drugs	Survivors (n = 170)	Non-survivors (n = 127)	*p*
Piperacillin/tazobactam	46 (27) (3 intermediates)	84 (66.1) (2 intermediates)	<0.001
Cefoperazone/sulbactam	81 (47.6) (3 intermediates)	101 (79.5) (2 intermediates)	<0.001
Cefazoline	93 (54.7)	105 (82.6)	<0.001
Ceftriaxone	82 (48.2) (2 intermediates)	102 (80.3)	<0.001
Ceftaxime	64 (37.6) (2 intermediates)	86 (67.7) (1 intermediate)	<0.001
Cefepime	58 (34.1) (1 intermediate)	81 (63.7)	<0.001
Amintronem	70 (41.1)	89 (70) (3 intermediates)	<0.001
Imipenem	43 (25.2) (1 intermediate)	79 (62.2)	<0.001
Ertapenem	42 (24.7) (2 intermediates)	82 (64.5)	<0.001
Tobramycin	50 (29.4) (19 intermediates)	68 (53.5) (17 intermediates)	<0.001
Amikacin	35 (20.5)	63 (49.6) (1 intermediate)	<0.001
Levofloxacin	59 (34.7) (3 intermediates)	83 (65.3) (4 intermediates)	<0.001
Ciprofloxacin	65 (38.2) (3 intermediates)	89 (70) (5 intermediates)	<0.001
Cotrimoxazole	73 (42.9)	72 (56.6)	<0.001
Polymyxin B	0 (1 intermediate)	0 (3 intermediates)	
Tigecycline	0 (2 intermediates)	0 (5 intermediates)	

**Table 5:** Comparison of the clinical data between 28-day survivors and non-survivors (n,%).

Characteristics	Survivors (n = 170)	Non-survivors (n = 127)	*p*
Mechanical ventilation^∫^	54 (31.7)	72 (56.6)	<0.001
Mechanical ventilation time^∭^	75 [29,189]	88 [36,178]	0.390
AKI^∫^	23 (13.5)	85 (66.9)	<0.001
CRRT^∫^	11 (6.4)	30 (23.6)	<0.001
Vasoactive agent^∫^	68 (40%)	77 (60.6)	<0.001
Hospitalization days^∭^	23 [14,37]	22 [11,36]	0.340
Transferred to ICU^∫^	88 (51.7)	95 (74.8)	<0.001
ICU hospitalization days^∭^	14 [7,25]	12 [6,19]	0.073
bacterial clearance^∫^	113 (66.4)	42 (33)	<0.001

^∫^Use Chi-Square Test to verify; ^∭^Use Mann-Whitney U Test to verify.

n sample size, AKI acute kidney injury, CRRT continuous renal replacement therapy.

In this study, binomial logistic regression was used to evaluate the impact of the above suspicious risk factors on the 28-day survival rate of the subjects. The studentized residual of seven patients was more than three times the standard deviation, but remained in the analysis. The final logistic model was statistically significant, with a χ^2^ = 63.772 and *p* < 0.001. The model can correctly classify 79.5% of the research objects. The sensitivity, specificity, positive predictive value, and negative predictive value of the model were 69.3, 87.1, 80, and 79.1%, respectively. Among the independent variables included in the model, tracheal intubation, respiratory failure, hypoalbuminemia, MODS, a SOFA score greater than 6, and CRKP were statistically significant. The specific information is shown in **Table 6**. Endotracheal intubation increased the risk of death by 2.986 times, and the mortality of patients with respiratory failure increased by 4.067 times. Patients with hypoalbuminemia had a 2.408-fold increased risk of death. The risk of death for patients with MODS was increased by 3.72 times. Patients with a SOFA score greater than 6 had a 3.757-fold increased risk of death compared to other patients (SOFA score of ≤6). With regard to resistance to carbapenem antibiotics, the risk of death in CRKP infections was 2.9 times higher than that in carbapenem-sensitive *K. pneumoniae* (CSKP) infections.

**Table 6:** Logistic regression analysis of risk factors of death in patients with KP bloodstream infections.

Characteristics	OR (95% CI)	*p*
Trachea cannula		
None	1	
Have	2.986 (1.484–6.009)	0.002
Respiratory failure		
None	1	
Have	4.067 (1.674–9.883)	0.002
Hypoalbuminemia		
None	1	
Have	2.408 (1.245–4.658)	0.009
MODS		
None	1	
Have	3.720 (1.206–11.479)	0.022
SOFA score		
≤6 points	1	
>6 points	3.757 (1.932–7.305)	<0.001
Characteristics of drug resistance		
CRKP	1	
CSKP	2.942 (1.585–5.461)	0.001

KP K. pneumoniae, OR odds ratio, CI Confidence interval, CVC central venous catheter, MODS multiple organ dysfunction syndrome, SOFA sequential organ failure assessment.

### Antimicrobial Therapy

Among the 297 patients with *K. pneumoniae* BSI, 35 patients were discharged and died before the drug sensitivity was reported, and the other patients used antibiotics for less than 48 hours. 262 patients with *K. pneumoniae* BSI who received antimicrobial therapy were divided into groups according to the results of the drug sensitivity test, as shown in **Table 7**. There were 97 patients with S-KP BSI, with an overall mortality rate of 21.6%. Among them, 81 patients were treated with single antibiotic (83.5%), and 16 patients were treated with a variety of antibiotics (16.5%). The combined use of antibiotics did not appear to improve the prognosis. Specifically, carbapenem antibiotics accounted for half of the monotherapy cases, while the remaining cases were treated with cefoperazone sulbactam (24.6%), piperacillin tazobactam (11%), and cephalosporins (11.3%). In the combined therapy cases, there were 10 cases in which carbapenem was used and four cases in which tigecycline was used, of which one case was treated with triple antibiotics. There were 87 patients with MDR-KP BSI, and the mortality rate (44.8%) was higher than that in the sensitive group. Among them, the number of cases treated with monotherapy was relatively less than that of the sensitive group (66.7%), which was still dominated by carbapenem, with a total of 31 patients. Among them, 18 cases died within 28 days, of which 13 cases (72.2%) were resistant to carbapenem, and only one case was resistant to carbapenem. Tigecycline was mainly used for combination treatment (65.5%), of which three cases were treated with triple antibiotics. The mortality rate in the single drug treatment group is higher than that in the combination treatment group, which deserves the attention of clinicians. There were 78 patients with XDR-KP BSI, with a mortality rate of 67.9%. Among the patients treated with antibiotic, 55.1% were treated with a single drug and the case fatality rate was 79%. The case fatality rate of the combined treatment group was 54.2%, of which a combined regimen based on tigecycline accounted for 80% of the cases. A total of six patients were treated with triple antibiotics, of which one patient died.

**Table 7:** Treatment and outcome of the 262 cases of KP bloodstream infection patients.

Treatment	n (%)	Mortality (%)
S-KP	97 (37)	21 (21.6)
Monotherapy	81 (83.5)	15 (18.5)
Carbapenem	41 (50.6)	9 (21.9)
Cefoperazone/sulbactam	20 (24.6)	4 (20)
Piperacillin/tazobactam	9 (11.1)	1 (11.1)
Ceftazidime	7 (8.6)	1 (14.2)
Cefepime	3 (3.7)	0
Cefminox	1 (1.2)	0
Combination therapy	16 (16.5)	6 (37.5)
Tigecycline + Carbapenem	2 (12.5)	1 (50)
Tigecycline + Piperacillin/tazobactam	1 (6.2)	1 (100)
Tigecycline + Carbapenem + Cefoperazone/sulbactam	1 (6.2)	0
Carbapenem + Cefoperazone/sulbactam	6 (37.5)	3 (50)
Carbapenem + Piperacillin/tazobactam	1 (6.2)	0
Carbapenem + Ceftazidime	2 (12.5)	1 (50)
Carbapenem + Cefminox	1 (6.2)	0
Piperacillin/tazobactam + Aminoglycosides	2 (12.5)	0
MDR-KP	87 (33.2)	39 (44.8)
Monotherapy	58 (66.7)	30 (51.7)
Carbapenem	31 (53.4)	18 (58)
Cefoperazone/sulbactam	10 (17.2)	4 (40)
Piperacillin/tazobactam	9 (15.5)	3 (33.3)
Cefepime	4 (6.8)	3 (75)
Ceftazidime	2 (3.4)	1 (50)
Cefotiam	2 (3.4)	1 (50)
Combination therapy	29 (33.3)	9 (31)
Tigecycline + Carbapenem	7 (24.1)	3 (42.8)
Tigecycline + Polymyxin B	1 (3.4)	0
Tigecycline + Piperacillin/tazobactam	2 (6.8)	1 (50)
Tigecycline + Aminoglycosides	5 (17.2)	1 (20)
Tigecycline + Cefoperazone/sulbactam	1 (3.4)	1 (100)
Tigecycline + Carbapenem + Cefoperazone/sulbactam	2 (6.8)	0
Tigecycline + Carbapenem + Aminoglycosides	1 (3.4)	0
Carbapenem + Polymyxin B	1 (3.4)	1 (100)
Carbapenem + Piperacillin/tazobactam	1 (3.4)	0
Carbapenem + Cefoperazone/sulbactam	4 (13.8)	1 (25)
Carbapenem + Ceftazidime	2 (6.8)	1 (50)
Piperacillin/tazobactam + Aminoglycosides	2 (6.8)	0
XDR-KP	78 (29.7)	53 (67.9)
Monotherapy	43 (55.1)	34 (79)
Carbapenem	25 (58.1)	22 (88)
Cefoperazone/sulbactam	10 (23.2)	9 (90)
Piperacillin/tazobactam	5 (11.6)	2 (40)
Cefepime	1 (2.3)	1 (100)
Ceftazidime	2 (4.6)	0
Combination therapy	35 (44.9)	19 (54.2)
Tigecycline + Carbapenem	11 (31.4)	7 (63.6)
Tigecycline + Polymyxin B	1 (2.8)	1 (100)
Tigecycline + Piperacillin/tazobactam	4 (11.4)	2 (50)
Tigecycline + Aminoglycosides	3 (8.5)	2 (66.7)
Tigecycline + Cefoperazone/sulbactam	4 (11.4)	3 (75)
Tigecycline + Carbapenem + Cefoperazone/sulbactam	3 (8.5)	0
Tigecycline + Carbapenem + Aminoglycosides	1 (2.8)	0
Tigecycline + Carbapenem + Polymyxin B	1 (2.8)	1 (100)
Carbapenem + Cefoperazone/sulbactam	4 (11.4)	3 (75)
Carbapenem + Aminoglycosides	1 (2.8)	0
Carbapenem + Cefepime	1 (2.8)	0
Carbapenem + Aminoglycosides + Cefoperazone/sulbactam	1 (2.8)	0

n sample size, S-KP sensitive K. pneumoniae, MDR-KP multidrug resistant K. pneumoniae, XDR-KP extensively drug resistant K. pneumoniae.

## Discussion

Since the beginning of this century, *K. pneumoniae* has been prevalent all over the world, and with increasing incidence. It has gained the attention of clinical workers: on the one hand, the increase in the detection rate of drug-resistant bacteria has made it more difficult for clinicians to choose antibiotics; on the other hand, drug-resistant *K. pneumoniae* infections have a high mortality rate (Rodriguez-Bano et al., 2018). It has been recommended that a combination of antibiotics be used to treat *K. pneumoniae* BSI, but there is still no further clinical evidence. Therefore, we collected the clinical data of patients with *K. pneumoniae* bacteremia and statistically analyzed the risk factors for drug-resistant bacterial infection, the risk factors for death, and the mortality of patients with different drug-resistant *K. pneumoniae*. The treatment plan was evaluated according to patient prognosis to improve the alertness of clinicians with regard to these cases and to provide guidance for the prevention of drug-resistant bacterial infections and reasonable treatment.

We collected and analyzed the clinical data of patients with *K. pneumoniae* BSI during the past five years. Of the 297 patients, S-KP accounted for only 38.4% of the total number of cases. Reviewing data on *K. pneumoniae* isolated from blood samples released in Greece, its resistance to carbapenem antibiotics ranged from less than 1% in 2001 to 30–60% in 2008. In 2006, researchers found that the reason for carbapenem resistance of this batch of CRKP was the presence of the bla_VIM-1_ gene (Souli et al., 2008; Vatopoulos, 2008). In 2008, a number of studies found the bla_KPC-2_ gene present in CRKP isolates and, at one time, there was an epidemic in two separate hospitals (Maltezou et al., 2009; Pournaras et al., 2009). Subsequently, CRKP was detected in the United States, Italy, Israel, and other countries around the world, one after another. In this study, we did not explore the molecular epidemiology, but put more emphasis on the clinician perspective. We compared the clinical characteristics of *K. pneumoniae* patients with different drug resistance and found that sex and age were not related to susceptibility to drug-resistant bacteria. Patients with recent ICU hospitalization history, recent surgical history, recent antibiotic use history, mechanical ventilation history, and hospitalization more than two weeks before the BSI diagnosis were more likely to be infected with drug-resistant bacteria. It is suggested that clinicians be more vigilant against the possibility of drug-resistant bacterial infections when facing the clinical manifestations of BSIs in these kinds of patients, such as fever, increased procalcitonin (PCT), or even shock. At the same time, several other possible risk factors should not be ruled out, such as patients with a recent history of invasive surgery, patients in the ICU ward, patients with diabetes, and patients with severe clinical symptoms. Mantzarlis et al. found that critically ill patients who received carbapenem or polymyxin antibiotics, patients who received mechanical ventilation, and patients who underwent invasive surgery were more likely to develop CRKP infection (Mantzarlis et al., 2013). However, all the patients included were ICU patients, and the samples included blood and airway secretions, as well as other infectious secretions. Freeman et al. found that patients with a history of ICU hospitalization and transplantation, including hematopoietic stem cell and solid organ transplant, had a higher risk of Extended-Spectrum β-Lactamases *K. pneumoniae* (ESBL-KP) infection (Freeman et al., 2014). However, fewer cases were included in this study, and there was no age limit. At the same time, patients who do not have ESBL-KP may also have carbapenem or even a variety of antibiotic-resistant bacteria. In this study, based on the results of the drug sensitivity test that clinicians are typically most concerned about, we analyzed the epidemiological characteristics, high risk factors, and prognosis for drug-resistant bacterial infections.

In this study, the 28-day all-cause mortality rate of the included patients was 42.8%. Patients were divided into groups according to the results of the different drug sensitivity tests. The results showed that the mortality rate of patients with S-KP bacteremia was 21.6%. The mortality rate of patients with MDR-KP bacteremia was 44.8%. And the mortality rate of patients with XDR-KP bacteremia was 67.9%. Studies by Hoxha et al. found that the 30-day attributable mortality rate of CRKP patients was 41%, and the risk of death was three times higher than that of CSKP patients (Hoxha et al., 2016). However, the inclusion population included all patients with *K. pneumoniae* infection, not limited to BSIs. Studies by Borer et al. found that the crude and attributable mortality rates of patients with drug-resistant *K. pneumoniae* were 71.9 and 50%, respectively (Borer et al., 2009). It was also found that the mortality rate of patients with MODS was 86.9% and that of patients with septic shock was 100%, which was significantly higher than that of patients with simple sepsis. Nearly 25% of the patients in the study conducted by Borer et al. were from nursing homes and, thus, pertained more to elderly patients. Only adult patients with clinically diagnosed BSI and only *K. pneumoniae* blood cultures were included in our study. The mortality data obtained are higher than that in previous studies. On the one hand, because of the large number of critically ill patients, a total of 38% of the patients had a history of living in ICU before hospitalization, and a total of 61.6% of the patients were admitted to ICU during hospitalization. On the other hand, because the outcome index uses a survival condition of 28 days after the BSI diagnosis, this study pertains to all-cause mortality rather than hospital mortality. Unlike other studies that include only CRKP or XDR-KP, this trial includes information on all patients with *K. pneumoniae* BSI with different drug sensitivity results.

We analyzed the patients with different clinical prognoses. Through univariate and multivariate analysis, it was clear that tracheal intubation, respiratory failure, hypoalbuminemia, MODS, a SOFA score greater than 6 points, and CRKP were independent risk factors for death within 28 days after diagnosis of *K. pneumoniae* infection. Additionally, patients with poor prognosis were more likely to get worse during the course of treatment, such as MODS, acute kidney injury (AKI), mechanical ventilation, and septic shock. The risk factors explored in this study were 28-day risk factors for death in all patients clinically and bacteriologically diagnosed with *K. pneumoniae* BSI. Mantzarlis et al. found that older patients and immunodeficient patients were independent risk factors for death in patients with CRKP infection in the ICU (Tian et al., 2019). Recent studies in southwest China have found that XDR-KP patients with solid tumors and septic shock are independent risk factors for death within 28 days. However, sputum was the main sample and only 13% of the samples were blood. At the same time, the positive culture results of the samples were not combined with the corresponding clinical symptoms. Falcone et al. showed that colistin resistance and intra-abdominal infection were independent risk factors for death in patients with CRKP septic shock (Falcone et al., 2016). Machuca et al. found that septic shock and admission to the ICU are independent risk factors for 30-day death in patients with CRKP, and combined anti-infective therapy can reduce the mortality of septic shock patients with CRKP bacteremia (Machuca et al., 2019). The detection rate of drug-resistant bacteria is increasing day by day, and the 2.9 times higher mortality rate of patients with CRKP infection versus CSKP patients highlights the importance of developing pre-control.

Compared to different treatment schemes, anti-infective combination therapy in patients with S-KP bacteremia did not demonstrate a significant benefit; whereas, monotherapy had a relatively lower cost and greater benefit. Although patients with MDR-KP bacteremia had a lower risk of monotherapy, more patients died in 28 days, and the two antibiotic regimens dominated by tigacycline or carbapenem appeared more frequently. The mortality rate of patients with XDR-KP bacteremia is very high. We found that the 28-day mortality rate of combined therapy was significantly lower than that of monotherapy. It is recommended to avoid anti-infective therapy with single antibiotics in the case of drug-resistant bacterial infections. For patients at high-risk for drug-resistant bacterial infection, if the possibility of Gram-negative bacteremia is considered, whether there is clinical benefit for combination anti-infective treatment to treat *K. pneumoniae* infection still needs to be confirmed by further clinical studies.

Our study has some limitations. First of all, we only counted the all-cause mortality of patients, but we included only patients with clear *K. pneumoniae* at the time of selection, excluding patients who died or were discharged within 24 hours after admission. Second, our sample size is small and retrospective observational studies were conducted. A larger sample is necessary for further randomized controlled trials, but taking into account the changes in drug use and the drug resistance spectrum over time, the patient data from the last five years were selected and no further review was conducted. Third, we did not compare the prognosis of treatment with binary and triple antibiotics. This was due to the fewer number of cases for combined use of three kinds of antibiotics. Thus, the reference value for comparison is small. Finally, and most importantly, our study does not represent the current epidemiological situation in China, let alone other countries. The specific distribution of drug resistance and the corresponding treatment plans still need to be further developed according to the local epidemiological characteristics.

## Conclusion

In short, our study found that the detection rate of drug-resistant bacteria was high in patients with *K. pneumoniae* BSI. Patients with a recent ICU hospitalization history, recent surgical history, recent antibiotic use history, and mechanical ventilation history were more likely to be infected with drug-resistant bacteria, and the susceptibility rate of the drug-resistant bacteria in patients who were hospitalized for more than two weeks before BSI was 4.34 times higher than that in patients with less than one week of hospitalization. The prognosis for drug-resistant bacterial infection is poor, especially XDR-KP infection, and the mortality rate is high. Combined treatment of more than two kinds of antibiotics is recommended in XDR-KP bacteremia patients.

## Data Availability Statement

The original contributions presented in the study are included in the article/supplementary materials. Further inquiries can be directed to the corresponding author.

## Ethics Statement

The studies involving human participants were reviewed and approved by the Scientific Research and Clinical Trial Ethics Committee of the First Affiliated Hospital of Zhengzhou University (Code 2020-KY-087). The ethics committee waived the requirement of written informed consent for participation.

## Author Contributions

SGZ and ZYY conceived and designed the study. LMS, LTS, and JLX participated in the collection of case data. SGZ and TWS conducted statistical analyses, and wrote the paper. All authors contributed to the article and approved the submitted version.

## Funding

This study was supported by the Overseas Research and Training Project of Talents of Health and Health Science and Technology in Henan Province in 2019 (grant no. HWYX2019020), the Scientific and Technological Innovation Leaders in Central Plains (grant no. 194200510017), Provincial Ministry Co-Construction Project from the Medical Scientific and Technological Research Program of Henan Province (grant no. SBGJ2018020), the “51282” Project Leaders of Scientific and Technological Innovative Talents from Health and Family Planning Commission in Henan Province (2016-32), Key scientific research project plan of colleges and universities in Henan Province (18B310039), and Science and Technology People-Benefit Project of Zheng Zhou (2019KJHM0001).

## Conflict of Interest

The authors declare that the research was conducted in the absence of any commercial or financial relationships that could be construed as a potential conflict of interest.

## Abbreviations

BSI, Bloodstream infection; MODS, Multiple organ dysfunction syndrome; CRKP, Carbapenem-resistant *K. pneumoniae*; ICU, Intensive care unit; XDR, Extensively drug- resistant; XDR-KP, Extensively drug-resistant *K. pneumoniae*; CDC, Centers for Disease Control and Prevention; CLABI, Central Line-Associated Bloodstream Infection; SCCM, Society of Critical Care Medicine; ESCIM, European Society of Intensive Care Medicine; MDR, Multidrug resistance; PDR, Pandrug resistance; EUCAST, European Committee on Antimicrobial Susceptibility Testing; CKD, Chronic kidney disease; APACHE, Acute Physiology and Chronic Health Evaluation; SOFA, Sequential Organ Failure Assessment; CVC, Central venous catheter; SD, Standard deviation; IQR, Interquartile range; OR, Odds ratio; CI, Confidence interval; S-KP, Sensitive *K. pneumoniae*; MDR-KP, Multidrug-resistant *K. pneumoniae*; CSKP, Carbapenem-sensitive *K. pneumoniae*; ESBL-KP, Extended-Spectrum β-Lactamases *K. pneumoniae*.
